# Harmonising artistic designs with private and collective notions of home: a focus group study of older persons’ experiences of art in residential care specialised in dementia care

**DOI:** 10.1186/s12877-025-05775-2

**Published:** 2025-02-19

**Authors:** Synneve Dahlin-Ivanoff, Ewa Wikström, Maja Gunn, Qarin Lood

**Affiliations:** 1 Institute of Neuroscience and Physiology, Department of Psychiatry and Neurochemistry, Sahlgrenska Academy, University of Gothenburg, Gothenburg, Sweden; 2 Centre for Ageing and Health– AgeCap, University of Gothenburg, Gothenburg, Sweden; 3 Centre for Health Governance (CHG), University of Gothenburg, Gothenburg, Sweden; 4 School of Business, Economics & Law, Department of Business Administration, University of Gothenburg, Gothenburg, Sweden; 5 HDK-Valand– Academy of Art and Design, University of Gothenburg, Gothenburg, Sweden; 6 Konstfack University of Arts, Crafts and Design, Stockholm, Sweden; 7 Centre for Person-Centred Care (GPCC), University of Gothenburg, Gothenburg, Sweden; 8 Institute of Neuroscience and Physiology, Department of Health and Rehabilitation, Sahlgrenska Academy, University of Gothenburg, Gothenburg, Sweden; 9 School of Nursing and Midwifery, Faculty of Health Sciences, La Trobe University, Melbourne, Australia

**Keywords:** Older people, Co-creation, Residential care facilities, Expressions of home, Art

## Abstract

**Background:**

Integrating art in residential care settings aims to create meaningful experiences and enhance the facility’s aesthetic as a home. However, the literature shows a gap, as the voices of older persons are largely absent. This study aimed to explore the views of older persons in residential care facilities specialised in dementia care regarding art and expressions of home. Older persons with cognitive impairments are frequently depicted as lacking meaningful contributions, limiting their opportunities to voice their opinions and influence their environment.

**Methods:**

This study used a qualitative explorative design with focus group method, allowing older persons with dementia to discuss their views of art in relation to expressions of home. Eleven older persons participated in four focus groups. This method encourages interaction between participants, shedding light on a collective understanding.

**Results:**

The study found that the residential care facility was perceived as a home by the participants, based on their descriptions of how both private and shared collective spaces in the facility fostered a dynamic sense of belonging. Art owned by the older persons often held personal value and they valued art for its intrinsic qualities, such as beauty, meaning, and expression. The perception of art differed between the private and collective spaces, and the discussions centered on aligning the artistic design with the participants’ notions of home to foster a socially engaging environment.

**Conclusion:**

Clearly defined private and collective spaces seem to be crucial for fostering self-determination and a sense of belonging for older persons living in residential care facilities specialised in dementia care. A balance between private and collective spaces boosted social engagement, with art playing a key role in reflecting older persons’ previous lives, shared interests and experiences, with co-creation of artistic design ensuring a dynamic environment.

**Supplementary Information:**

The online version contains supplementary material available at 10.1186/s12877-025-05775-2.

## Background

Art has the power to evoke emotions, provide pleasure, and bring vitality to people’s lives, and it can play a crucial role in illness prevention, health promotion, and treatment throughout life [1]. Various forms of art—such as painting and drawing—have been used in dementia and psychiatric care with older persons, but evidence on the impact of art on older persons living in residential care facilities is still emerging [2, 3]. One aim with integrating art in everyday life in residential care settings has been to create meaningful personal experiences to enhance the aesthetic sense of the facility as a home [4]. Building on these findings, this study aimed to explore the views older persons who live in residential care facilities have of art in relation to expressions of home, with the goal to lay the foundation for the artistic design of a new residential care facility in western Sweden.

When designing residential care facilities, special attention must be placed on the older persons living there and their views of the environments they live in. Older persons living in residential care facilities often experience cognitive impairments and morbidity that affect their daily lives in various ways. In Sweden, a person typically needs to be over the age of 65 years to be granted an apartment in a residential care facility and have been professionally assessed as needing intermittent care and support that cannot be provided in ordinary housing [5, 6]. Unfortunatley, persons living with dementia are ofen faced by stigma, being unfairly portrayed in the media as if they do not have thoughts and desires that are important or worth paying attention to. This kind of depiction can lead to misunderstandings and negative attitudes towards them [7]. In relation to art, researchers [8, 9] describe that co-creation is an important tool as it promotes creativity, innovation, and diversity. Co-creation means that different actors, in this case artists and older persons in residential care facilities, participate in a joint process where they explore and express their ideas, experiences and visions. Through co-creation, art can become more relevant, engaging, and inclusive for everyone involved [8, 9]. Therefore, this study was inspired by the Capability Approach to highlight the voices of older persons living in residential care facilities in relation to the artistic design of these facilities. As described by the philosopher Martha Nussbaum [10, 11] the Capability Approach has come to put focus on human rights and dignity, and is deeply rooted in the idea of justice. With focus on what persons are able to do and be, the capability approach emphasises the importance of providing persons with the freedom to pursue their own goals and aspirations. This approach encourages inclusive practices that take into account the diverse capabilities of all participants, fostering a more equitable and participatory environment [10, 11]. Within this study, the Capability Approach was applied to take the participants’ desires and needs as the point of departure in the research process, focusing on justice and freedom of choice and a view of all human beings as capable to influence their everyday lives and living environment.

Artistically designed physical environments have the potential to support both individual persons’ health and well-being and the society as a whole, suggesting that health, well-being and the environment are symbiotic [12]. For example, benefits of art, creativity, and culture are associated with supportive environments both indoors and outdoors, offering an innovative approach to providing opportunities for restorative and regenerative activities among older persons [13, 14]. The powerful impact of the physical environment on well-being is also gaining recognition with the term “Healing Built Environment”, used to describe healthcare buildings that (1) reduce the stress levels for all healthcare building users, and (2) promote health benefits for persons in need of healthcare [15]. Groot et al. [16] conclude that an artistically designed environment involving those directly involved highlights the intrinsic value of artistic participation. Further, artistic design can have a positive impact on older persons, immersing them in joyful moments, personal challenges, and deep connections, promoting their well-being [16].

Despite the benefits of older persons participating in artistic design in their environments, their voices are largely missing from existing research on artistic activities and ageing [17, 18]. Much of the existing studies on artistic activites have been conducted from the perspective of professionals, service providers or researchers who may not fully understand the realities and challenges of older persons [17]. For example, Chacur et al. [17] show that research on artistic activities with older persons has progressively increased over the last ten years, but most research has focused on the benefits of participation in art activities [11]. A recent scoping review [19] exploring the use of art activities with older persons living in residential care facilities shows that art activities can be used to create a homelike atmosphere within these facilitites, helping older persons to feel comfortable and connected to their living environment, promoting well-being. Art activities allow older persons to express their personal histories and identities, which can evoke a sense of home and belonging, reduce anxiety and promote a sense of security [19]. Nevertheless, there is a paucity of research that includes the perspectives and voices of older persons themselves.

Previous research has shown that art can serve as a valuable instrument for enhancing the democratic fabric of our lived environment and its artistic expressions, and have a powerful role in nurturing social bonds, enabling self-expression, and promoting well-being among persons with cognitive impairment living in residential care facilities [16, 19, 20]. However, previous empirical research that involves older persons in art activities tends to focus on the positive outcomes of their participation in the activities, rather than their subjective experiences and feelings [18], which are crucial to understand in order to meet their unique and diverse needs [21]. Addressing this knowledge gap, this study is part of a research project with the overall aim to artistically design a residential care facility by collaborating in the planning, construction and furnishing process and to evaluate the importance of art and design for older persons in terms of social engagement and sustainable living environments. The project is part of GoCo Health Innovation City, which brings different worlds, fields, and people together for inspiring collaboration and co-innovation. More specifically, the project is a multidisciplinary collaboration between researchers at the Centre for Aging and Health (AgeCap) at the University of Gothenburg and the company “Vectura Fastigheter AB” within the construction of community properties, such as residential care facilities. The project is being conducted in two phases: (1) exploration of the planning, constructing, and furnishing process and; (2) evaluation of the artistic process. This study was carried out as part of the planning, construction and furnishing process as a first step to co-create the residential care facility environment through artistic measures. As such, the purpose of this study was to address shortcomings in previous research by ensuring the voices of older persons are heard. The aim was to explore what views older persons who live in residential care facilities specialised in dementia care have of art in relation to expressions of home.

## Methods

The study had an explorative design, applying focus group methodology [22, 23]. This method allowed the older persons to make their voices heard by meeting each other and discussing their views of artistic design in relation to expressions of home. We strove for shared experiences and focused on variation in the collective understanding that emerged from the group discussions, stemming from a qualitative approach based on a social constructivist approach [24, 25]. The Consolidated Criteria for Reporting Qualitative Research [26], were applied during the writing of the manuscript (see supplementary material).

### Study context

The study was carried out in a residential care facility specialised in dementia care. The facility accommodated up to 64 persons with varying needs of care and support in everyday life. In compliance with Swedish regulations for residential care facilities for older persons, all persons living in the facility had access to continuous care from direct care staff, primarily assistant nurses with upper secondary care education [6]. Allied health professionals, registered nurses, and physicians are also available as needed for each person.

### Participants

Convenience sampling was applied [27], meaning that all persons assessed by the residential care facility staff as capable of providing informed consent were eligible to participate in the study. They were invited to the study by the general manager and staff. Ethical considerations, such as informed consent and respecting the independence and dignity of participants, were prioritised through a person-centred approach to research [28]. Ensuring that participants fully understand and retain the consent information was crucial, especially given that memory issues can complicate this process. The age range of the participants was 83–90 years. A total of eleven persons, two men and nine women, took part in the study. Out of these persons, two were actively engaged in two separate focus group discussions each.

### Procedure

A total of four focus groups were conducted. One group had two participants, one group had three participants, and two groups had four participants each (*n* = 11, as two persons participated in two groups each). The focus group discussions were carried out by the first and last author (SDI and QL) in the conference room at the residential care facility, and the sessions began with the group leader (SDI or QL) informing the older persons about the study and its purpose. Both group leaders had previous experience from conducting focus group studies, and from working with frail older persons with and without cognitive impairments. In one of the focus group, a staff member was in the room to provide support to one of the participants. The staff member did not participate in the discussions.

The group leader’s task was to pose questions to deepen the discussions with support from the visual images of art, and to ensure that all persons were given a chance to speak, identifying common elements in the discussions and posing general questions followed by more specific questions. Two key questions were formulated. The discussions were initiated with dialogues on the notion of home, based on the first key question: “How do you view your living environment as your home, particularly in relation to the presence and influence of art?”. This first question was followed by discussions based on the second key question: “How can art be expressed in residential care facilities?”. To deepen the discussions and explore older persons’ views on art and its expression in a residential care facility, we used images to guide the discussions. These images represented different types of art, such as paintings, photographs, textiles, sculptures, and applied art. The images were displayed on a large screen and discussed with the older persons. Striving for equal opportunities for older persons living in residential care facilities to make their voices heard, the discussions were built on mutual respect to facilitate each person’s participation. This is especially important when involving older persons with different degrees of cognitive impairment. Each focus group lasted about 1.5 h or less and were digitally audio-recorded.

### Analysis

The audio-recordings were professionally transcribed verbatim by a transcription firm and reviewed for accuracy in transcription by the first author. The analysis followed the focus group analysis method developed by Kreuger and Casey [23] and was primarily conducted by the first and last author. The third and fourth author were also involved in the analysis, but at a later stage. The method aims to understand collective, common experiences, emphasising the insights that emerge from the interactions among focus group participants [23, 24, 29]. It was crucial to keep the raw data accessible long enough to grasp the material’s meaning. Therefore, the analysis was conducted in Swedish until a final formulation of themes and sub-themes was completed. In the analysis, the researcher explored how the target group collectively understood the study´s focus, as the meaning can only be analysed within the context of the discussion [23, 24]. To become familiar with, and gain an understanding of, the content of the material in its context, the first step in the analysis was listening to the audio recordings several times, guided by the aim of the study. At this stage, the working material was still in the form of raw data. The transcript of each group discussion was then read carefully and independently to get an overall sense of the data. Next, sections relevant to the research topic were identified and sorted into different themes. The aim of the study guided our comprehension of the contextual meaning of the material. Themes that emerged from our review of the raw data were defined, and descriptive statements synthesising, abstracting, and conceptualising the data were constructed. The last step was to summarise the categorised raw data, combined with an interpretative step aiming to provide understanding. The purpose of the analysis was to find patterns that can be visualised and transformed into easily understandable material. The data analysis process was iterative; that is, each step was initially conducted by the first author separately and then discussed with the last author, followed by a discussion with all authors to triangulate the interpretative step to enhance the credibility [23, 24].

### Rigour

We attended to the several key elements to ensure the thoroughness and trustworthiness of the research process and findings [30]. First, we carefully planned for the focus group discussions to be coherent by aligning the aim, conduct, analysis and presentation of findings. To ensure credibility, we also planned for the role of the moderator(s), and how they would interact with the involved persons to ensure that all voices were heard and acknowledged [24, 29]. Second, we practiced reflexivity by recording the discussions and taking notes on non-verbal cues, such as participants leaving the room or showing signs of disengagement. Third, the analysis was systematic, conducted in accordance by an established method [23] to ensure that the interpretations were based on what the older persons said. In addition, the analysis was kept in the original language until a final thematic structure had been established, to avoid interpretation errors. The authors who moderated the groups (SDI and QL) were responsible for the analysis, which was verified by the other members of the research group (EW and MG) to minimise researcher bias. Member checking was not conducted as it is not recommended for focus groups [31]. However, we made every attempt to ensure that the persons involved could add to the understanding by summing up the discussions during each session. For dependability, quotations are used to signify the participants discussions around certain topics. Fictive names are used to give life to the quotations without compromising the participants’ confidentiality.

## Findings

### Harmonising artistic design with private and collective notions of home

Although many persons cannot take care of their home at the residential care facility, it was important that it is not just a residence, but a home. In fact, the residential care facility was perceived as two homes: the private home (the apartment) and the collective home (the dining room and living room). Both notions of home were considered important for the older persons to enjoy their stay in the residential care facility, but the role of art was experienced somewhat differently in the private home and the collective home. The discussions evolved around how to harmonise the artistic design of the facility, with the older persons’ private and collective notions of home creating a dynamic whole for well-being and social engagement. This is further described in two themes, both with subthemes (see Table 1 for an overview of the findings).

**Table 1:** Overview of the findings

Harmonising artistic design with private and collective notions of home	
*The private home– to decide for oneself*	*The collective home– a meeting place*
Our home is the staff’s workplace	The collective environment as a social function
Art in my home– a picture of myself	Art as a picture of the collective
Art is beauty, meaning, and experiences	

### The private home - to decide for oneself

The older persons described the importance of deciding for themselves in their own home. It was important to feel that they have their own private home with their own door as a boundary to the collective home. This meant that staff and other older persons living in the residential care facility should knock on the door and wait for an answer before entering the older persons’ apartment. As a resident, one should have the right to choose when to let someone in, which means that others should respect that if no one answers, they should not enter but come back at another time. It also means that they have chosen the art in the private home themselves, and as a person, it marks that this is my home, and I decide here. Art was perceived by the older persons as unique for each person and place. This is highlighted through three sub-themes: *Our home is the staff’s workplace*,* Art in my home - a picture of myself*, and *Art is beauty*,* meaning*,* and experiences.*

### Our home is the staff’s workplace

The residential care facility served as both the home for older persons and the workplace for staff. This dual purpose fostered a dynamic sense of security by balancing the needs of the older persons with the practical requirements of staff. However this also meant that the older persons could not expect the same level of personal freedom to rearrange furniture or make changes as they did in their previous homes, since the facility must remain accessible for cleaning and for staff to provide necessary care and support. Art could be incorporated into the home’s design to be both aesthetic and functional and collaboration between the older persons and staff was described as crucial to find a compromise that satisfied everyone. This required balancing the desires and needs of the older persons, with the practical needs of the staff’s work environment. Staff must recognise that moving into a residential care facility represents a significant change for the older persons, who have left their previous homes, such as a house or villa. Understanding and empathy from the staff were described as essential to help the older persons adjust to this new living situation. We have chosen a quotation from focus group two to illustrate the meaning of the sub-theme.

Moderator (SDI): *Yes*,* and what do you think makes it a home?*

Harry: *An important thing is that we have something that is our own room or our own apartment… But I think it is very important that we feel and know that this is our home. Then we also feel better. And of course*,* we are all different*,* and therefore I have noticed that many*,* after a while*,* see it as their home and start decorating and arranging things so that they feel at home. But I think that is a very important part. And it is also important for the managers here. They say that it is a care home*,* but it is still our home*,* no matter how you look at it… Then I think the staff has been very good… I believe they have chosen staff who like older people. So*,* it has been a very good support.*

Moderator (SDI): *Do you have any thoughts*,* Anita? About what makes it feel like a home?*

Anita: *Yes*,* I am very satisfied. I really can’t complain. Everything is kind and helpful. I can’t say more.*

Moderator (SDI): *And how do you think about your own apartment in relation to the other rooms here? Does everything feel like it is your home?*

Harry: *Yes*,* well… There has been a bit of a discussion because if you have your own home*,* you are responsible… Yes*,* then you do as you please. But this is a residence. So you kind of… You can’t do entirely as you want. You can’t bring in large items. But I think if you express your wishes*,* you can meet halfway maybe. You shouldn’t just say no*,* here we are in charge.*

### Art in my home - a picture of myself

The art that the older persons have in their own homes should have some form of personal value. The older persons found it more enjoyable with art that they either recognise or that depicts something. They had chosen the art themselves, to mark what is their private home where they decide, including the choice of art. In the private home, the older persons wanted art with a personal value that meant a lot to them as a person. In this context, they defined drawings made by children and grandchildren as art they liked in their homes. It could also be art by well-known artists or art painted by relatives or friends. Photographs were also seen as art, forming important memories and a picture of their lives and how it was in the past. What all older persons had in common was that the art they had in their private homes in some way showed who they are and what life they have lived, and that all art has a very personal value. This is illustrated by a quotation from focus group four:

Anna: *I have an old ornament that is almost 80 years old.*

Moderator (QL): *Oh.*

Tage: *And I made a little… a little troll in kindergarten. And it has followed me my whole… my whole life. And it stands in a place of honor at home. And I always pat it on the head before I… the troll and say good night.*

[laughter]

Tage: *That’s the kind of ideas you have. And then I sleep well.*

Anna: *What if you forget it one night?*

Tage: *No*,* I never do. It stands by my bed where I lie.*

Anna: *Oh*,* it stands by the bed*,* yes?*

Tage: *Yes*,* and it’s so easy to reach out my… good night to you*,* little troll.*

Anna: *Yes*,* yes. Yes*,* it is.*

Moderator (QL): *Do you take it with you when you travel somewhere?*

Tage: *Yes*,* of course.*

### Art is beauty, meaning, and experiences

Art could evoke strong emotions and was perceived differently by different persons. A painting might be perceived as scary by one person but not by another. Similarly, art could be seen as beautiful even if it appears gloomy and dark. Art could also feel too personal if it stirs thoughts and feelings that become overwhelming and unpleasant. The motif in a painting was described as crucial because it conveys a message. Art should communicate something, and viewers should be able to grasp what the artwork represents. It should not be overly abstract; instead, the painting should resonate with the viewer, conveying meaning. This applies to both abstract art, which expresses emotions and ideas, and realistic art, which portrays the tangible world. Sometimes, a motif could evoke memories from the past, related to people or things. Each person’s taste and experiences influence their perception of art. Whether you like a painting depends on what it says to you—it breathes life into the canvas. Additionally, everyone has their favourite colour, and the choice of colour adds excitement to the artwork. The choice of paintings is illustrated by a quotation from focus group three.

Moderator (SDI): *What do you think of the painting?*

Gunn: *I think it’s nice. I like the colors*,* and it’s mine.*

Britt: *It’s yours.*

Gunn: *Yes. If I take it now*,* it’s mine. And that’s important to me.*

Inga-Britt: *Candy cane*,* that’s what I think.*

Gunn: *Yes*,* but why… It’s not entirely wrong when you say that. I like it when there’s a bit of… a bit of excitement in it. It doesn’t have to be what you see every day and square*,* but it can be a bit random like that. Then it’s a bit more fun*,* I think.*

Moderator (QL): *Britt*,* did you think it was nice?*

Britt: *Yes*,* I did.*

Moderator (QL): *Would you like to have it in your room?*

Britt: *Yes*,* I think I could have it.*

## The collective home- a meeting place

The collective home was described as a common space where the older persons met each other and socialised. The dining room and living room were considered the most important rooms, seen as an extension of one’s own home and a meeting place. If it did not exist, the older person would spend much of their time in their apartments and there would be no communal living. It was in the dining room and living room where the older persons met and talked. If they wanted to be alone, then went into their private home. Art in the collective home was also viewed as a joy for the eye, and as a socialising function. It could be completely different than in one’s private home, to represent the interests of all persons living there and give an image of the collective. What was most important in the collective home is described in two sub-themes: *The collective environment as a social function*, and *Art as a picture of the collective.*

### The collective environment as a social function

The common areas were regarded as key gathering spots. Art within these collective environments was appreciated not only for its visual appeal but also for its role in encouraging social interactions. As an older person living in a residential care facility, they wanted to be respected and listened to, despite being frail, having memory problems, and reduced cognitive function. The older persons expressed that they did not want to be ashamed for forgetting things, for being confused, or for making mistakes. Many who had moved into the facility had done so because of loneliness and the facility should provide opportunities to go out and socialise so that one does not become isolated. Just by going out from their apartment, they have company. In addition, the environment provided freedom for the older persons by offering a place for relaxation, activities, socialising, experiencing, and learning new things. It was also important that there were activities that could be done together, and art could play an important role here. The staff also had an important role, and it was considered important that staff listen and understand that they age as persons. This is illustrated by a quotation from focus group one:

Moderator (SDI): *Do you like going to the dining room?*

Harry: *Yes*,* I think it is nice. It is really the o my place where we all meet*,* since everyone has their own room. Dining together is a nice way of socialising.*

Sonja: *It would be kind of boring and the food would not taste as food if you sat alone and dined.*

Harry: *We also have the advantage that if someone is sick or cannot go out*,* they get their food in their room.*

Moderator (SDI): *Is there a common area where you can sit down and watch TV or talk?*

Harry: *There is no common area*,* but I think it’s enough that we meet three times a day*,* during meals.*

Sonja: *One always has something to talk about when you meet in the dining room.*

Lena: *Mmm*,* yes.*

Harry: *It is an own living space*,* and you have your own apartment. If you want to meet people*,* you go out*,* and I think that’s very good.*

Lena: *Yes.*

### Art– a picture of the collective

There are many different persons living in a residential care facility, with different opinions and views, and there are as many opinions as there are persons. It was therefore suggested to change art in the collective home (dining room and living room) at certain time intervals, and that everyone should be given the opportunity to decide which art should be there. Because the collective changes over time, such co-creation could create a living art environment that represents those who live in the facility at that time. The older persons were happy to participate and give their opinion on art that should hang in the collective home. It was important that the art reflected both the era in which the persons who live in the facility have lived, and the knowledge and experience they have of art from before. Portraits were perceived as less appropriate in the collective home, unless they were portraits of persons that everyone could relate to. The older persons’ views of art as a picture of the collective is illustrated by a quotation from focus group two:

Moderator (QL): *If you think about sculptures*,* are they something you would want here?*

Harry: *Yes*,* yes*,* sculptures are always beautiful. They are small pieces of artwork. But this group here (shown on image)*,* it is very difficult to make such group*,* but here they have been very clear about it. But then the thing is also that if you have such a group*,* you might get tired of it. But you can change it*,* and have them for a certain period of time. That is the advantage with things that you can take a way and bring back and…*.

Moderator (QL): *Where would you have sculptures if you should have them here?*

Harry: *it should be in the big room of course. But then you have to*,* I think… Since we are so many here*,* we have many opinions. Then you might do it in such a way that for a period*,* maybe half a year or a year*,* you have some people involved. In the next half year*,* you bring in another group to share their opinions. And then you can alternate so that everyone gets a chance to participate.*

Moderator (QL): *Yes*,* that is a very good suggestion. What do you think about sculptures Lena?*

Lena: *Yes*,* maybe the red one. It is very nice. Simple*,* but I think that is nice. Very nice.*

## Discussion

This study aimed to explore how older persons who live in residential care facilities specialised in dementia care view art in relation to expressions of home. Key findings underscore the need for a dynamic and inclusive home environment, respecting diverse preferences and needs. Art in the private and collective homes was described as immensely valuable, mirroring the older persons’ identities and life experiences, helping them to have a sense of home. They appreciated art for its beauty, meaning, and experiential qualities and desired art that reflects their collective interests and era, fostering a sense of community and social connections. Essentially, the residential care facility should feel like a home, balancing private and collective spaces to enhance well-being and social engagement. Art plays a crucial role in this process, with different experiences and preferences for art in private and collective spaces. Importantly, art activities can align with person-centered approaches as described by Durocher et al. [19], by tailoring the choice of activity and the level of involvement to match the individual preferences, capabilities, and needs of older persons.

The study reveals an important finding: residential care facilities, which serve as both the older person’s home and the staff’s workplace, can be perceived as “home” by the older persons living there. This dual perception fosters a dynamic sense of security. Previous research [32] have showed that having access to the things one needs most and having someone close by in case one needs help generates a feeling of security, suggesting that older persons value the sense of security provided by residential care facilities. In a Norwegian study [33], older persons described feeling at home as having a sense of security within the residential care facility and a single room where they could retreat. The participants wanted greater possibilities for meaningful relations and appreciated that the residential care facility was similar to their previous homes and wished for opportunities to continue with the activities they did in their former home [33].

Another study [34] interestingly showed that older persons spend most of their time in the common living room. However, this space blurs the boundary between the public and private spheres, unlike the clear boundaries typically associated with a traditional home. The researchers [34] described that one way to realise the idea of the residential care facilities as a home could be to define the living room as a more clearly public area and to give the older person a chance to develop a more private lifestyle by switching between their private rooms and a general shared living room. The results showed how important it was for older persons to have their own home with their own door as a boundary to the collective home which marks that this is my home and I decide here [34]. However to truly become a home it is not enough to be merely connected to the residential care facility and its immediate environment. In line with our findings, Malcolm et al. [35] describe that involvement in on-site activities is a significant prerequisite of becoming at home [35]. This highlights the significance of balancing the private home and the collective home to enhance well-being and social engagement. Our results showed that common areas were regarded as key gathering spots. Art within these collective environments was appreciated not only for its visual appeal but also for its role in encouraging social interactions. The descriptions of the collective environment as a social function describes the communal spaces as critical meeting points. Simply by leaving their apartments, the older persons find company, and art plays a crucial role in this process, with different experiences and preferences for art in private and collective spaces [36]. Staff are integral to this context, and they need to listen actively and understand the experiences and challenges faced by older persons to create a supportive and empathetic environment. As underscored in the literature on person-centredness [21, 37] it is important to recognise each person as a unique person with distinct abilities, resources, and life history. It involves the building of trust between those in need of health and social care and staff providing these services. To achieve person-centred care, joint decisions must be made in partnership [21, 37]. This means considering the knowledge and experiences of both the older person and the staff. Rather than focusing solely on diagnoses and impairments, attention should be directed toward resources and needs. Creating positive relationships based on mutual respect is essential for promoting self-determination [38]. Self-determination refers to the ability of a person to make decisions based on their own free will. It encompasses having control over one’s life and understanding one’s ethical and legal rights [39]. The goal to enhance the opportunities for each older person to live a dignified and meaningful life. In relation to our findings, person-centredness could be applied to acknowledge the older persons’ wishes that the choice of art in the collective home should involve them to represent the interests of all persons and give an image of the collective. It was also important for the older persons that the art reflected both the era in which the persons who live in the accommodation have lived, and the knowledge and experience they have of art from before.

Another significant finding in this study is that the art owned by older persons in their homes often holds personal value. This art, in some way, reflects who they are and the life they have lived. Hagvedt et al. [40] described five ways in which viewers engage with art and the extent to which we are captivated by it. These include (1) our emotional response to the art, (2) whether it is perceived as aesthetically pleasing, (3) how the craftsmanship is appreciated, (4) if it stimulates our intellect, and finally (5) how innovative and creative the art is perceived to be. In Hagvedt et al.‘s [40] description of how viewers engage with art the personal value is not described, i.e., that it holds personal significance for the person as it represents memories from their life. Research [32, 41] shows that if you do not have any memories associated with your home, it does not feel like home. It is important not to underestimate the significance of these memories for older persons. This was also reflected in our study, illustrating the value of art for evoking memories of the past and telling the older persons’ life stories. Jackson [42] further indicates that the presence of meaningful objects is a strategy to provide a sense of continuity. Being surrounded by objects filled with memories enables older persons to combine the past and the present in their daily experience and to find life meaningful. Memories are not just passive recollections of the past, but active constructions of the present and the future. They can help older persons maintain their cognitive, emotional, and social well-being, and enrich their lives with joy, comfort, and meaning [41, 42].

Our results also showed that the older persons in this study valued art for its intrinsic qualities, such as beauty, meaning, and expression. Rather than seeing art as a simple tool or intermediary, the older people saw it as an end in itself. This individual view of art differs from some of the dominant views in the literature, which emphasise the therapeutic or cognitive benefits of art for older persons [17, 43]. Chacur [17] and Lee [44] highlight that while many studies focus on the positive effects of older people’s participation in artistic activities on health and well-being, there is a lack of research examining the experiences of older people themselves, regardless of whether these experiences lead to measurable health benefits or not [17, 44]. This means that while we know a lot about how artistic activities can benefit older persons, we know less about how the older persons themselves experience and engage in creativity and art in their later years. Essentially, our findings highlight the need for more research on the subjective, personal experiences of older persons in relation to art, beyond just the measurable health benefits.

Because the collective changes over time, co-creation of the environment is considered a tool for a living art environment to create an artistic environment that represents the older persons interests and experiences at present. Unfortunately, their valuable insights and perspectives are often overlooked, due to ageism and can lead to experiences of exclusion, as described in previous literature [45]. To address this, researchers must actively challenge existing power imbalances and involve older persons in research to ensure that their voices are heard, not only in studies but also when designing policies that can truly benefit them.

### Scientific implications

Including older persons in research and policy-making is crucial to combat ageism and ensure their rights and needs are met. This inclusion can lead to more age-friendly policies that better address their specific needs and challenges. It not only results in more informed and just decision-making but also empowers older persons by valuing their insights and experiences. Co-creation in art within residential care facilities fosters creativity, innovation, and diversity, making artistic spaces more relevant and inclusive. By involving older persons in the artistic process, these spaces reflect their collective interests and life experiences, enhancing their sense of belonging and engagement. This approach aligns with the capability approach, which emphasises empowerment and recognition of the value of diverse contributions. As such, co-creation with older persons living in residential care facilities can elevate the artistic design process beyond mere aesthetics, making it a dynamic and participatory experience that celebrates the unique abilities and histories of the older persons. Furthermore, addressing ageism through inclusive practices in research and policy design ensures that older persons’ voices are heard and their contributions acknowledged. This can lead to person-centred environments that are not only more supportive and engaging but also more just and equitable.

### Method discussion

When carrying out focus group studies with persons who have cognitive impairments it is crucial to be aware of that they may find it challenging to express themselves verbally or understand complex subjects [8]. Research [46, 47] on engaging persons with dementia in studies emphasised the importance of ethical considerations, such as informed consent and the need to respect the independence and dignity of participants. The necessity for flexible research methodologies is highlighted, to adapt to the unique challenges posed by cognitive impairments. Previous research [46, 47] also underscore the value of person-centred approaches that focus on the strengths and abilities of persons with dementia rather than their limitations. These findings collectively underscore the importance of ethical sensitivity, meaningful engagement, and methodological flexibility in research involving people with dementia [46, 47]. We have incorporated these findings into our focus groups by ensuring interactions are respectful and empathetic, obtaining informed consent, and prioritising participants’ comfort. We created a valued and inclusive environment using open-ended questions to encourage discussion and adapted methods to meet participants’ needs, such as using visual aids and allowing more time for responses. By fostering a supportive atmosphere where participants feel safe sharing their views, we ensure that focus groups are effective, respectful, and provide valuable insights while honoring participants’ dignity and independency.

Four focus groups were conducted in this study. While this limited number of groups might impact information power [48] and the applicability of the results, it is important to consider the context. Some experts [22, 49] argue that four to five focus groups are sufficient, especially when dealing with specific target groups [22, 49], as is the case in this study Additionally, the number of older persons in some of the focus groups was low. Previous literature does not provide strict criteria but suggests a range of four to 12 participants for focus groups [46]. According to other researchers, the ideal group size is four to eight people [24, 45]. Research [50] highlights the importance of limiting the number of participants in focus groups, especially when it comes to vulnerable groups. The authors emphasise that smaller groups can create a safer environment and enable deeper discussions, which is particularly important when participants may feel vulnerable [50], as in this study due to the context of living in a residential care facility.

Recruiting people with dementia for research presents significant challenges. Memory issues are a major factor, affecting participants’ ability to recall details about the study, including its purpose, procedures, and their own consent to participate [51]. Keeping track of appointments and follow-up visits can be difficult, leading to missed sessions and inconsistent participation. These factors were evident in this study, highlighting the complexities involved and justifying the number of focus groups conducted. This version acknowledges the potential limitations while also providing a strong rationale for the number of focus groups based on the specific challenges of working with people with dementia. However, despite potential limitations, the study benefits from vibrant and dynamic interaction among the older persons. Their active engagement seemed to play a more crucial role in shaping the outcome than the sheer number of participants [29, 52]. When dealing with older persons with cognitive impairment as meaningful interactions, person-centered care, and tailored activities can make a difference [53].

In this study, both group leaders of the focus group had experience from working with persons with cognitive impairments, and all older persons that the residential care facility staff deemed capable of giving informed consent were invited to participate in the focus groups. In a prior study [54], we paired photographs with individual dialogues to explore the meanings and significance that the older persons attributed to the images. This method was beneficial as the photographs assisted the older persons in recalling memories and expressing what they considered important. This strategy also proved to be a vital aid for the discussion in this study. Focus group discussions acted as a venue for generating new insights through interpersonal interaction and we employed art images to improve communication conditions.

The older persons in this study seemed to appreciate the opportunity to take part in the focus groups, resulting in fruitful discussions in which the older persons shared their views—both positive and negative. Negative views have been found to be more easily expressed when having a common ground with others [23]. It was stressed that the older persons were experts, and they were able to express their views on a relevant subject; this gave them a strong voice, which they appreciated. Previous research [24] has also shown that being grouped with others with the same experiences, being able to discuss things with people who understand, and knowing that you are not the only one with a particular experience create a feeling of sharing.

The collective nature of focus groups can, according to the literature [24] empower the participants, and focus groups are especially useful for engaging people with limited power and influence, such as, for example, older persons with cognitive impairment [24]. Feeling a sense of fellowship with others in similar situations may encourage research participants to express things that would not be discussed otherwise [55]. The awareness of sharing similar experiences can make participants realise that their views are legitimate and valid [23, 29], which seemed to be the case in the present study.

## Conclusion

Residential care facilities was perceived as “home” by the older persons, fostering a sense of security through access to resources and support. Continuity of activities and meaningful relationships from previous homes, along with art activities tailored to individual preferences, enhanced the older persons’ well-being. Clearly defining private and collective spaces was essential for self-determination and belonging. Active involvement in on-site activities and balancing private and collective spaces enhanced social engagement, with art playing a significant role. Communal spaces fostered social connections and a sense of belonging, supported by empathetic staff. Recognising each person’s unique abilities and life history, and promoting self-determination, is therefore crucial. The older persons desired art in collective homes to reflect collective interests and experiences, with co-creation ensuring a dynamic artistic environment. This way, the results focus on the perception of residential care facilities and the personal value of art, while the conclusions emphasise the importance of security, continuity, and the role of communal spaces and activities in enhancing well-being and social engagement.

## Electronic supplementary material

Below is the link to the electronic supplementary material.

Supplementary Material 1

## Data Availability

The data produced and analysed in this study are not accessible to the public. This is because the participants were assured of confidentiality when they gave their informed consent. However, de-identified data can be provided by the corresponding author upon reasonable request for review purposes. These data will be securely stored at the University of Gothenburg, Sweden, for a period of 10 years from the date of publication. It is important to note that all data are subject to the Swedish Public Access to Information and Secrecy Act, and each request for access will undergo a confidentiality assessment.
